# Evaluation of the Educational Value, Reliability, and Quality of Vaginally-Assisted Natural Orifice Transluminal Endoscopic Surgery (v-NOTES) Videos on YouTube

**DOI:** 10.7759/cureus.85244

**Published:** 2025-06-02

**Authors:** Isa Temur

**Affiliations:** 1 Department of Obstetrics and Gynecology, Nigde Omer Halisdemir University, Faculty of Medicine, Nigde, TUR

**Keywords:** gqs, jama, mdiscern, video quality, youtube

## Abstract

Objective

This study aims to evaluate the quality, reliability, and educational usability of vaginally-assisted natural orifice transluminal endoscopic surgery (v-NOTES) videos on YouTube (Google Inc., Mountain View, CA).

Methods

This cross-sectional study analyzed 58 videos retrieved from a YouTube search using the keyword "v-NOTES." Quality and reliability assessments were conducted using the Journal of the American Medical Association (JAMA) score (0 to four), the modified DISCERN score, the Global Quality Scale (GQS; 0 to five), and the Usefulness Score (US). Video popularity was evaluated using metrics such as viewer engagement, number of likes, like ratio, view count, view rate, and the Video Power Index (VPI).

Results

Of the evaluated surgical videos, 39 (67.2%) covered hysterectomy, while 19 (32.8%) featured other procedures. Doctors uploaded 44 (75.9%) of the videos, and medical companies shared 14 (24.1%). Animated content was present in 16 (27.6%) of videos, while 42 (72.4%) were non-animated. Among 58 analyzed videos, median quality scores were mDISCERN: three, JAMA: two, and GQS: two. Low-quality videos accounted for 43.1% based on the US. Median video characteristics were duration 330 seconds, views 1300, view rate 1.5, likes 23.5, VPI 0.3, and engagement score 1.6. No significant difference in quality and reliability was found between videos from doctors and medical companies. However, animated videos had higher median mDISCERN, JAMA, and GQS scores, with a significant difference in usefulness scores (p = 0.008).

Conclusion

This study assessed the quality, reliability, and educational value of YouTube videos on v-NOTES, revealing that despite the abundance of content, overall quality and reliability remain inadequate.

## Introduction

Originally introduced in 2004, natural orifice transluminal endoscopic surgery (NOTES) is a minimally invasive technique that has been refined in recent years. This approach allows surgeons to access internal structures through natural body openings such as the mouth, anus, vagina, or urethra, eliminating the need for abdominal wall incisions [1,2]. Vaginally-assisted natural orifice transluminal endoscopic surgery (v-NOTES) is an innovative technique that integrates the benefits of endoscopic methods with vaginal surgical procedures and has been adopted for a range of gynecological applications [3]. Interest in v-NOTES has grown in recent years, driven by its advantages, such as improved visualization of pelvic structures, reduced operative time, superior cosmetic outcomes without scarring, and the elimination of trocar-related complications [4]. Consequently, v-NOTES is gaining widespread attention in gynecology, particularly for procedures such as adnexectomy, hysterectomy, myomectomy, sacrocolpopexy, and, more recently, its applications in oncologic surgery [5,6].

In the last decade, social media has become a major platform for accessing healthcare-related information. Studies indicate that approximately 80% of adults in the United States have turned to the internet for health information [7]. YouTube (Google Inc., Mountain View, CA), a widely available video-sharing platform, ranks among the top three most visited websites worldwide. With over four billion videos viewed each day and more than 500 hours of new content uploaded every minute, it serves as a major source of online information [8]. YouTube is gaining popularity as a platform where users can access, share, and engage in discussions about health-related information. While social media offers the advantage of timely updates, it also poses risks due to the lack of content regulation and the absence of a peer-review process, which may lead to misleading or inappropriate information [9,10].

Although studies evaluating the quality of healthcare-related content on YouTube are increasing, no research to date has specifically assessed the quality and educational value of v-NOTES surgical videos on the platform. To address this gap, the present study aimed to evaluate the usability, reliability, and educational quality of YouTube videos on v-NOTES for both healthcare professionals and patients.

## Materials and methods

Study design

A systematic search was conducted on YouTube on December 1, 2024, using the keywords “vaginally assisted natural orifice transluminal endoscopic surgery,” “v-note surgery,” and “vNOTES.” The search was performed in incognito mode with cleared search history and cookies to reduce the influence of personalized algorithms on the search results. To ensure the relevance and timeliness of the content, only videos uploaded within the last five years (between December 1, 2019, and December 1, 2024) were considered. A total of 58 videos meeting the inclusion criteria were selected for analysis. Institutional Ethics Committee approval was not obtained due to no patient data being used.

Exclusion criteria included (1) videos unrelated to the v-NOTES procedure, (2) content featuring other types of surgery, (3) videos exceeding 30 minutes or incomplete, (4) content in languages other than English, and (5) videos with poor audiovisual quality. All videos were independently evaluated by a single experienced reviewer (T.T.) using predefined assessment criteria to ensure consistency and standardization in the evaluation process. For each video, the following data were recorded: title, source (e.g., physician, institution, or commercial), content type (informative, operative, or promotional), category (animated or non-animated), duration (seconds), number of views, time since upload (years), and the number of likes and dislikes. The use of a single evaluator is acknowledged as a potential limitation due to the risk of subjective bias. This limitation is further addressed in the discussion, with a recommendation that future studies incorporate multiple independent reviewers to enhance inter-rater reliability and methodological robustness.

Assessment of video quality and reliability

Using these data, the viewer engagement index [(number of likes - number of dislikes) / total number of views × 100%] [11], view rate (number of views/number of days since upload), like ratio [likes / (likes + dislikes) × 100], and video power index (VPI) [(view rate × like ratio) / 100] [12] were calculated. The videos' reliability, completeness, and quality were evaluated using the modified DISCERN (mDISCERN) tool, the Global Quality Scale (GQS), and the Journal of the American Medical Association (JAMA) score. Furthermore, their usefulness for physician education was assessed using the Usefulness Score (US) [12,13].

mDISCERN Tool

The mDISCERN tool is a validated measure and is one of the most commonly used instruments for evaluating electronic healthcare information. The mDISCERN score (ranging from zero to five) was developed to provide an objective assessment of the quality of videos. The scale is composed of five questions: Are the objectives clearly defined and attainable? Are the information sources credible and trustworthy? Is the content presented in a fair and unbiased manner? Are additional resources provided for patients? Are any areas of uncertainty acknowledged? Each "yes" response corresponds to one point, indicating a positive aspect of the content [12].

JAMA Benchmark Criteria

The JAMA scoring system is a general and objective method for assessing the credibility of online videos and resources [12]. It evaluates content based on four distinct criteria, each worth one point, resulting in a total score ranging from 0 to four. A score of four signifies high reliability and accuracy, while a score of 0 indicates low reliability and accuracy. Criteria definition: Authorship: the names, qualifications, and institutional affiliations of the authors and contributors should be clearly stated. Attribution: proper credit must be given by explicitly listing copyright details, references, and sources for the content. Currency: the publication date and any updates or revisions to the content should be clearly indicated. Disclosure: conflicts of interest, funding, sponsorship, advertising, support, and video ownership should be fully disclosed.

GQS

GQS is used to evaluate the quality of non-specific educational content. It evaluates the educational usefulness of video content for patients using five specific criteria. Each criterion is given one point, with a maximum possible score of five, representing the highest level of educational quality [13]. Quality score definitions: Poor quality: not suitable for patient education; Low quality: provides limited value for patients, as it contains only partial information; Moderate quality and clarity: somewhat useful for patients; lacks essential topics but includes some relevant details. Good quality and clarity: beneficial for patients, covering most key topics. Excellent quality and clarity: highly informative and valuable for patient education.

US

The US for physicians [13] evaluates eight key categories in YouTube videos: definition, indications, contraindications, advantages, related procedures, complications, postoperative care, prognosis, and survival. Each category was assigned one point, resulting in a total score ranging from 0 to eight. A score between 0 and two indicates poor video content with misleading information. A score between three and five represents moderate-quality content, conveying a positive message about v-NOTES but lacking depth in certain areas. A score between six and eight reflects detailed, valid, and accurate content that provides comprehensive and highly useful information for physicians.

Statistical analysis

The Shapiro-Wilk test was used to assess whether the variables met the assumption of normality. Descriptive statistics for the videos and scores were presented as count (n), percentage, median, minimum, and maximum values. For comparisons between two groups, the Mann-Whitney U test was used when the assumption of normality was not met. The Yates-corrected chi-square test was applied for the comparison of categorical variables. The relationship between video characteristics and scores, which did not follow a normal distribution, was evaluated using Spearman’s correlation test. All statistical analyses were performed using IBM SPSS Statistics software, version 25.0 (IBM Corp., Armonk, NY). A p-value <0.05 (α) was considered statistically significant.

## Results

A total of 58 videos were included in the study. Among the surgical videos, 67.2% (n=39) were related to hysterectomy procedures, while 32.8% (n=19) were categorized as other types of surgeries; 75.9% (n=44) of the videos were uploaded by doctors, whereas 24.1% (n=14) were uploaded by medical professionals. Additionally, 27.6% (n=16) of the videos were animated, while 72.4% (n=42) were non-animated. Descriptive statistics regarding video characteristics and quality scores are presented in Table 1. The median values were calculated as follows: video length = 330 seconds, number of views = 1,300, view rate = 1.5, number of likes = 23.5, VPI = 0.3, and viewer engagement = 1.6. In the US category, 43.1% (n=25) of the videos were rated as poor, 37.9% (n=22) as moderate, and 19% (n=11) as excellent. The median quality scores were three for mDISCERN, two for JAMA, and two for GQS (Table 1).

**Table 1:** Descriptive statistics for features and quality scores of videos VPI: Video Power Index; mDISCERN: modified DISCERN score; JAMA: Journal of the American Medical Association; GQS: Global Quality Scale

Variables	n	%
Name of the surgery		
Hysterectomy	39	67.2
Other	19	32.8
Video category		
Animation	16	27.6
No animation	42	72.4
Time elapsed since installation (year)		
1	7	12.1
2	12	20.7
3	15	25.9
4	18	31.0
5	6	10.3
Loading source		
Doctor	44	75.9
Medical	14	24.1
Usefulness Score		
Poor	25	43.1
Moderate	22	37.9
Excellent	11	19.0
	Median	Min.-Max.
Video length (seconds)	330	90-3600
Number of views	1300	100-35000
View rate	1.5	0.2-41
Number of likes	23.5	0-135
VPI	0.3	0.1-55.3
Audience interaction	1.6	0.01-12.5
mDISCERN	3	1-5
JAMA	2	0-3
GQS	2	1-5

Table 2 displays the relationships between video characteristics and quality scores. A weak but positive and statistically significant correlation was found between video length (in seconds) and mDISCERN, JAMA, GQS, and US (rs=0.399, p=0.002; rs=0.327, p=0.012; rs=0.382, p=0.003; rs=0.327, p=0.012, respectively). A weak but positive and statistically significant correlation was identified between the number of days since upload and the number of likes (rs=0.327, p=0.012). The video view count exhibited a strong positive correlation with the view rate and VPI, while showing a moderate positive correlation with the number of likes (rs=0.901, p<0.001; rs=0.890, p<0.001; rs=0.690, p<0.001, respectively). However, viewer engagement exhibited a strong negative correlation with view count (rs=-0.780, p<0.001).

**Table 2:** Correlations between video features and quality scores **p<0.01; *p<0.05 significance level (pairwise); rs: Spearman's rank correlation coefficient; VPI: Video Power Index; mDISCERN: modified DISCERN score; JAMA: Journal of the American Medical Association; GQS: Global Quality Scale; US: Usefulness Score

n=58		Video length (seconds)	Number of days	Number of views	View rate	Number of likes	VPI	Audience interaction	mDISCERN	JAMA	GQS	US
Video length (seconds)	r_s_ p	1.000	-0.013 0.926	-0.014 0.916	0.064 0.631	0.121 0.364	0.092 0.493	0.0730 0.587	0.399** 0.002	0.327* 0.012	0.382** 0.003	0.327* 0.012
Number of days	r_s_ p	-	1.000	0.241 0.069	-0.122 0.360	0.327* 0.012	0.071 0.599	-0.1240 0.352	0.023 0.863	0.220 0.097	0.085 0.526	-0.020 0.881
Number of views	r_s_ p	-	-	1.000	0.901** <0.001	0.690** <0.001	0.890** <0.001	-0.780** <0.001	0.049 0.719	0.172 0.196	0.031 0.816	0.067 0.616
View rate	r_s_ p	-	-	-	1.000	0.599** <0.001	0.894** <0.001	-0.715** <0.001	0.083 0.536	0.115 0.389	0.030 0.821	0.099 0.461
Number of likes	r_s_ p	-		-	-	1.000	0.819** <0.001	-0.2310 0.082	0.199 0.135	0.418** 0.001	0.269* 0.041	0.299* 0.022
VPI	r_s_ p	-	-	-	-	-	1.000	-0.604** <0.001	0.154 0.248	0,288* 0.028	0.123 0.356	0.242 0.067
Audience interaction	r_s_ p	-	-	-	-	-	-	1.000	0.116 0.387	0.077 0.565	0.212 0.110	0.137 0.304
mDISCERN	r_s_ p	-	-	-	-	-	-	-	1.000	0.793** <0.001	0.849** <0.001	0.802** <0.001
JAMA	r_s_ p	-	-	-	-	-	-	-	-	1.000	0.828** <0.001	0.806** <0.001
GQS	r_s_ p	-	-	-	-	-	-	-	-	-	1.000	0.787** <0.001
US	r_s_ p	-	-	-	-	-	-	-	-	-	-	1.000

Regarding video view rate, there was a moderate positive correlation with the number of likes and a strong positive correlation with VPI (rs=0.599, p<0.001; rs=0.894, p<0.001, respectively). Additionally, viewer engagement showed a strong negative correlation with view rate (rs=-0.715, p<0.001). A strong positive correlation was found between the number of likes and VPI (rs=0.819, p<0.001), while a weak positive correlation was observed between the number of likes and mDISCERN, JAMA, GQS, and US (rs=0.418, p=0.001; rs=0.269, p=0.041; rs=0.299, p=0.022, respectively). Moreover, the mDISCERN score showed a strong positive correlation with JAMA, GQS, and US (rs=0.793, p<0.001; rs=0.849, p<0.001; rs=0.802, p<0.001, respectively). Similarly, the JAMA score had a strong positive correlation with GQS and US (rs=0.828, p<0.001; rs=0.806, p<0.001, respectively). Finally, there was a strong positive correlation between GQS and US (rs=0.787, p<0.001).

A statistically significant difference was found between animated and non-animated videos in terms of the median values of mDISCERN, JAMA, and GQS scores (p < 0.001), while no significant difference was observed for other video characteristics (Table 3). The median values of mDISCERN, JAMA, and GQS scores were higher in animated videos compared to non-animated videos. Additionally, a statistically significant difference was found between US categories and animated versus non-animated videos (p=0.008). Among animated videos, 50% were classified as having a moderate US, whereas among non-animated videos, only 33.3% had a moderate US.

**Table 3:** Comparison of scores and video-related characteristics according to whether the video is animated or not p<0.05 significance level; variables are given as median (minimum-maximum and n (%) values. a: Mann-Whitney U test, b: Pearson's chi-square test; VPI: Video Power Index; mDISCERN: modified DISCERN score; JAMA: Journal of the American Medical Association; GQS: Global Quality Scale

Variables	Animation (n=16)	No animation (n=42)	p-value
Video length (seconds)	380(105-1500)	315(90-3600)	0.465^a^
Time after loading (days)	1050(350-1860)	1085(340-1860)	0.537^a^
Number of views	2800(100-35000)	1200(200-30000)	0.217^a^
Imaging rate	2(0.2-33.3)	1.25(0.3-41)	0.115^a^
Number of likes	31(0-113)	22(4-135)	0.292^a^
VPI	0.6(0.1-8.7)	0.2(0.1-55.3)	0.085^a^
Audience interaction	9.21(0.01-12.5)	1.86(0.15-4.88)	0.439^a^
mDISCERN	4(2-5)	2(1-5)	<0.001^a^
JAMA	2.5(1-3)	1(0-3)	<0.001^a^
GQS	4(2-5)	2(1-5)	0.001^a^
Usefulness Score			
Poor	2(12.5)	23(54.8)	-
Moderate	8(50)	14(33.3)	0.008^b^
Excellent	6(37.5)	5(11.9)	-

A statistically significant difference was found between videos uploaded by doctors and those uploaded by medical companies in terms of the median values for view count, view rate, like count, VPI, and viewer engagement (p<0.001, p=0.001, p=0.019, respectively), while no significant difference was observed for other characteristics and quality scores (Table 4). Videos uploaded by medical companies had higher median values for view count, view rate, like count, and VPI compared to those uploaded by doctors. Conversely, the median viewer engagement value was higher for videos uploaded by doctors.

**Table 4:** Comparison of scores and video-related characteristics according to the source of the video p<0.05 significance level; variables are given as median (minimum-maximum) and n (%) values. a: Mann-Whitney U test; b: Pearson's chi-square test; VPI: Video Power Index; mDISCERN: modified DISCERN score; JAMA: Journal of the American Medical Association; GQS: Global Quality Scale

Variables	Doctor (n=44)	Medical companies (n=14)	p-value
Video length (seconds)	335(90-3600)	285(105-1780)	0.326^a^
Time after loading (days)	1065(340-1860)	1050(360-1860)	0.899^a^
Number of views	1200(100-13003)	4000(900-35000)	<0.001^a^
Imaging rate	1(0.2-10.4)	3.25(0.6-41)	<0.001
Number of likes	18(3-132)	42.5(0-135)	0.001^a^
VPI	0.2(0.1-11.8)	1.3(0.2-55.3)	<0.001^a^
Audience interaction	1.96(0.15-5)	1.2(0.01-12.5)	0.019^a^
mDISCERN	3(1-5)	3(1-5)	0.395^a^
JAMA	1(0-3)	2.5(0-3)	0.066^a^
GQS	2(1-5)	3.5(1-5)	0.252^a^
Usefulness Score			
Poor	20(45.5)	5(35.7)	>0.999^b^
Moderate	16(36.4)	6(42.9)	0.678^b^
Excellent	8(18.1)	3(21.5)	0.732^b^

## Discussion

Despite offering vast access to information, the internet poses challenges regarding content accuracy, as most online medical information is not peer-reviewed. Today, many patients use the internet to explore their health conditions and treatment options, with YouTube becoming one of the most frequently utilized platforms for this purpose [14]. Rapp et al. reported that YouTube is the leading platform for visual medical and surgical information [15]. However, its open-access nature, allowing unrestricted video uploads, raises concerns about reliability and contributes to information pollution [16]. Thus, evaluating the quality of medical content on YouTube has gained increasing importance [13]. Although several scoring systems have been developed to assess the quality and reliability of health-related online content, few studies have specifically focused on the educational value of YouTube videos related to v-NOTES surgery. In this study, we applied validated tools such as mDISCERN, JAMA, US, and GQS to assess both the quality and usefulness of such videos.

Our analysis showed that the median scores were three for mDISCERN, two for JAMA, and two for GQS, with 43.1% of videos categorized as low quality based on US. The significant positive correlations among mDISCERN, JAMA, GQS, and US indicate internal consistency across these assessment tools. These results suggest that videos considered useful for patient education are also valuable for physician training, highlighting the need for higher-quality, more comprehensive educational content. Physicians uploaded 75% of the analyzed videos, a proportion similar to findings reported by Brooks et al. [17], though other studies have noted lower rates [18,19]. Prior research generally associates physician-generated content with higher quality scores [19-22]. Nevertheless, our study found no statistically significant difference in quality scores between physician-uploaded videos and those from other sources. This may reflect variations in presentation clarity, as physician-generated content often contains complex medical terminology, limiting accessibility for general audiences [23].

Consistent with prior studies [20,24,25], our findings indicated that videos from non-physician medical sources received more views, likes, and higher VPI scores. Nonetheless, median viewer engagement was greater for physician-uploaded videos, suggesting continued relevance within professional or informed audiences. Of the 58 videos evaluated, 27.5% were animations and 72.5% were real surgical footage. Animated content was primarily uploaded by health-related channels. Similarly, Akpolat et al. reported a comparable proportion of animated videos in their analysis of YouTube content on Bankart lesions [26]. Cassidy et al. also highlighted that patients perceive animated videos as more useful, emphasizing the need for such content [9].

In our study, animated videos scored significantly higher in mDISCERN, JAMA, GQS, and US (p = 0.008) compared to real footage. However, no significant differences were observed in VPI, video length, view count, or like rate between the two formats. The popularity of YouTube videos is generally assessed based on metrics such as the number of views and the like ratio. In our study, a strong positive correlation was found between the number of likes and the VPI, while DISCERN, JAMA, GQS, and US scores showed a weak but statistically significant positive association with the number of likes. However, this relationship between popularity and educational quality is not always consistent. For instance, Kuru et al. reported a negative correlation between video quality and the number of likes, suggesting that high-quality videos may not necessarily achieve widespread popularity [20]. Similarly, Koçyiğit et al. found no significant differences in views, likes, or comments across different quality categories [27]. Other studies have also demonstrated that lower-quality content can attract more likes and views [28,29].

This observed inconsistency between video popularity and educational quality is a critical finding. It underscores that metrics such as views and likes do not reliably indicate the accuracy, reliability, or usefulness of content, especially from the perspective of patient audiences. Therefore, patients should be cautioned against interpreting popularity as a proxy for informational quality, and efforts should be made to direct them toward validated and expert-reviewed resources.

Strengths and limitations

This study has several limitations. First, as YouTube is a dynamic platform with continuously changing content, the findings reflect only a snapshot in time and may not remain valid as new videos are uploaded or removed. Second, the inclusion was limited to English-language videos, restricting the generalizability of results to non-English-speaking populations. Although the search was conducted in incognito mode with cleared cache and cookies to minimize algorithmic personalization, YouTube search results may still vary depending on users’ geographic location and device settings. Additionally, differences in keyword usage (e.g., “vaginally assisted natural orifice transluminal endoscopic surgery,” “v-NOTES surgery,” “vNOTES”) may influence which videos are retrieved, potentially affecting the comprehensiveness of the sample. Another significant limitation is the use of a single evaluator for video assessment. Although validated instruments such as DISCERN and GQS were employed, these tools inherently involve subjective interpretation. Relying on one reviewer may introduce bias, potentially compromising objectivity and consistency. Future research should include multiple independent reviewers and employ inter-rater reliability analysis to improve methodological rigor and reproducibility. Finally, the relatively small sample size limits the study's ability to generalize findings to the broader population of v-NOTES-related videos available online. As a result, the conclusions drawn may not fully reflect the overall quality and educational value of all relevant content on YouTube.

## Conclusions

This study evaluated the quality, reliability, and educational value of YouTube videos related to v-NOTES gynecological surgery. The findings revealed that although a large number of videos are available, their overall quality is generally low, and popularity metrics do not reflect educational value. Therefore, YouTube should not be regarded as a reliable source of information, and patients are advised to consult healthcare professionals. To enhance the quality of online medical education, the promotion of peer-reviewed content produced by medical experts is essential.
